# Prospective Cohort Study of Childhood-Onset Stargardt Disease: Fundus Autofluorescence Imaging, Progression, Comparison with Adult-Onset Disease, and Disease Symmetry

**DOI:** 10.1016/j.ajo.2019.11.008

**Published:** 2020-03

**Authors:** Michalis Georgiou, Thomas Kane, Preena Tanna, Zaina Bouzia, Navjit Singh, Angelos Kalitzeos, Rupert W. Strauss, Kaoru Fujinami, Michel Michaelides

**Affiliations:** a University College London Institute of Ophthalmology, University College London, London, United Kingdom; b Moorfields Eye Hospital, London, United Kingdom; c Department of Ophthalmology, Johannes Kepler University, Linz, Austria; d Department of Ophthalmology, Medical University Graz, Graz, Austria; e Laboratory of Visual Physiology, Division of Vision Research, National Institute of Sensory Organs, National Hospital Organization Tokyo Medical Center, Tokyo, Japan; f Department of Ophthalmology, Keio University School of Medicine, Tokyo, Japan

## Abstract

**Purpose:**

To determine the reliability and repeatability of quantitative evaluation of areas of decreased autofluorescence (DAF) from fundus autofluorescence (FAF) images and track disease progression in children with Stargardt disease (STGD1), and to investigate clinical and genotype correlations, disease symmetry, and intrafamilial variability.

**Design:**

Prospective cohort study.

**Methods:**

Children and adults with molecularly confirmed STGD1 (n = 90) underwent longitudinal FAF imaging with subsequent semiautomated measurement of the area of DAF and calculation of the annual rate of progression. The age of disease onset was recorded for all subjects, as well as the electroretinography (ERG) group at baseline (n = 86). Patients were grouped for analysis based on the age at baseline and age of onset, into children (n = 56), adults with childhood-onset STGD1 (n = 15), and adults with adult-onset (n = 19). Fifty FAF images were selected randomly and analyzed by 2 observers to evaluate repeatability and reproducibility. Differences between groups, interocular symmetry, genotype–phenotype correlations, and intrafamilial variability were also investigated both for baseline measurements as well as progression rates. We measured visual acuity, molecular genetics, ERG group, FAF metrics, and their correlations.

**Results:**

The mean age of onset ± SD was 9.6 ± 3.4 years for childhood-onset (n = 71) and 28.3 ± 7.8 years for adult-onset STGD1 (n = 19). The intra- and interobserver reliability of DAF quantification was excellent (intraclass correlation coefficients 0.995 and 0.987, respectively). DAF area was symmetric between eyes and the mean rate of progression (SD) was 0.69 (0.72), 0.78 (0.48), and 0.40 (0.36) mm^2^/year for children, adults with childhood-onset, and adults with adult-onset disease, respectively. Patients belonging to a group 3 ERG phenotype (generalized cone and rod dysfunction) had a significantly greater progression rate. Limited intrafamilial variability was observed.

**Conclusions:**

This is the first large prospective study of FAF in a cohort of molecularly confirmed children with STGD1. DAF area quantification was highly reliable and may thereby serve as a robust structural endpoint. A high rate of progression was observed in childhood-onset disease, making this subtype of STGD1 ideally suited to be considered for prioritization in clinical trials.

## Highlights

• First large prospective study of fundus autofluorescence in a cohort of molecularly confirmed children with STGD1.
• Fundus autofluorescence is a robust structural outcome measure in children with Stargardt disease.
• A high rate of progression was observed in childhood-onset disease, making this subtype of STGD1 ideally suited to be considered for prioritization in clinical trials.

Stargardt disease (STGD1; OMIM 248200) is the most common inherited macular dystrophy with a prevalence of 1 in 8000 to 10,000 individuals.^1^, ^2^, ^3^, ^4^, ^5^, ^6^, ^7^ Onset is most frequent in childhood, where patients present with bilateral central visual loss and characteristic macular atrophy, often with yellow-white flecks at the level of the retinal pigment epithelium (RPE) at the posterior pole.^1^^,^^2^^,^^5^^,^^8^^,^^9^ STGD1 has an autosomal recessive mode of inheritance associated with disease-causing sequence variants in the adenosine triphosphate–binding cassette, subfamily A, member 4 (*ABCA4*) gene (MIM; 601691).^1^^,^^10^, ^11^, ^12^, ^13^ *ABCA4* encodes the retinal-specific transmembrane protein, which is localized to the rim of rod and cone outer segment discs and is involved in the active transport of retinoids.^14^, ^15^, ^16^, ^17^, ^18^, ^19^, ^20^, ^21^, ^22^, ^23^ The lack of or inefficient removal of *N*-retinylidene-phosphatidylethanolamine from photoreceptor outer segments caused by *ABCA4* dysfunction/mislocalization ultimately results in toxic levels of phosphatidylpyridinium bisretinoid (A2PE) in photoreceptor membranes.^23^, ^24^, ^25^ A2PE is hydrolyzed to form the highly toxic metabolite A2E, which accumulates as a component of lipofuscin in RPE cells. Over time, A2E-associated cytotoxicity/phototoxicity is believed to cause photoreceptor degeneration, RPE dysfunction, and ultimately RPE loss, which are hallmarks of STGD1.^26^^,^^27^

Fundus autofluorescence (FAF) is a noninvasive imaging modality that uses the autofluorescent properties of lipofuscin and related fluorophores, providing valuable information on the distribution of lipofuscin in the RPE.^9^ Lipofuscin levels are determined largely by the activity of ABCA4 in the photoreceptors; failure or reduced function leads to increased bisretinoids formation.^26^^,^^27^ Increased lipofuscin levels have been correlated with photoreceptor loss.^26^ The abnormal accumulation of lipofuscin, the presence of active and resorbed flecks, and RPE atrophy lead to a characteristic appearance on FAF imaging in STGD1: low autofluorescence signals in photoreceptor and RPE atrophy and foci with low or high signals caused by flecks.^25^^,^^28^, ^29^, ^30^ A previous cross-sectional study has shown that FAF patterns relate to functional abnormalities^28^ and 2 longitudinal studies have shown an association between atrophy enlargement and electrophysiological findings.^29^^,^^31^ Fujinami and associates characterized FAF subtypes, demonstrating that progression of atrophy was influenced by 2 background FAF patterns (homogeneous and heterogeneous) and that multiple atrophic lesions at the posterior pole were associated with more rapid functional deterioration.^9^ Their cohort consisted of predominantly adults, including both childhood-onset and adult-onset STGD1.

Previous studies of STGD1 progression based on area measurements of RPE atrophy on FAF or photoreceptor loss on optical coherence tomography (OCT) range from 0.28 mm^2^/year to 1.58 mm^2^/year.^9^^,^^29^^,^^31^, ^32^, ^33^, ^34^, ^35^ The wide range in progression rates found in the above studies may likely be explained by differences in the study populations—including the age of disease onset, age of presentation, and genetic background. In addition, only 3 studies report repeatability and reliability metrics,^9^^,^^35^^,^^36^ so the precision of the measurements is not known in the majority of studies, which could also contribute to the differences in reported disease progression rates. The ongoing and upcoming clinical trials in STGD1 create a prominent need for further investigation of robust anatomic outcomes, both for patient stratification as well as for monitoring response to treatment. Quantifying measurements on FAF in children would be valuable in understanding the natural history of STGD1 in young patients and help determine whether children have better retinal potential for therapeutic intervention, as well as whether FAF metrics can serve as a structural endpoint. Despite increasing evidence that childhood-onset STGD1 belongs to the severe end of the spectrum of *ABCA4*-associated retinal phenotypes,^4^^,^^7^^,^^36^^,^^37^ the rate of progression using FAF imaging in a large cohort of molecularly proven children has not been investigated.

The purpose of this study was to investigate FAF imaging qualitatively and quantitatively over a clinically significant follow-up period in a large, well characterized cohort of children with genetically confirmed STGD1, and to explore potential correlations of progression rate with age of disease onset, disease duration, genotype, and baseline area of atrophy and electrophysiological group. The measurement reliability and repeatability were established, with disease symmetry and intrafamilial variability also investigated.

## Methods

This prospective observational study adhered to the tenets of the Declaration of Helsinki and was approved by the Moorfields Eye Hospital Ethics Committee. Informed consent and assent were obtained from guardians and children, respectively, and consent from adult patients was obtained before entering the study.

### Patients

Ninety patients with STGD1 were recruited from a single tertiary referral center (Moorfields Eye Hospital, London, United Kingdom) and had annual visits over 6 years. In 17 patients, previously acquired data with the same protocol were used to extend the follow-up time. All patients had their detailed medical history documented, including age of disease onset. For the purpose of this study, we divided our cohort into 3 subgroups based on age at baseline investigation and the age of disease onset. Age of disease onset was defined as the age of the first symptom(s), which was defined as any vision disease–related complaint. Childhood-onset and adult-onset disease was defined as having disease onset before or after 17 years of age, respectively. All patients examined at baseline at <17 years of age were defined as children and had childhood-onset STGD1 (n = 56). All patients ≥17 years of age at baseline were analyzed separately as adults, and are further subdivided into adult-onset STGD1 if they were symptomatic at ≥17 years of age (n = 15), or as adults with childhood-onset STGD1 if they were symptomatic before 17 years of age (n = 19). The duration of the disease was calculated as the difference between age at onset and age at the last follow-up examination when FAF imaging was obtained.

### Genotype Classification

All recruited patients were molecularly confirmed and had either ≥2 likely disease-causing variants in *ABCA4*, or ≥1 likely disease-causing *ABCA4* variant and a typical phenotype. In keeping with previous studies, segregation analysis was possible in a limited number of cases because of the unavailability of other family samples. We classified patients into 4 groups depending on the number and type of the identified variants, as previously described.^3^^,^^4^^,^^9^ The description of genotype group classification is summarized in Table 1. Null variants were those that would be expected to affect splicing or to introduce a premature truncating codon in the protein if translated.^9^

**Table 1:** Grading Systems

Genotype Classification	ERG Group	FAF Pattern
Class A: Multiple null variants Class B: One null variant Class C: Multiple missense variants Class D: One missense variant and/or uncertain effect variant(s)	Group 1: Dysfunction confined to the macula Group 2: Macular and generalized cone system dysfunction Group 3: Macular and generalized cone and rod system dysfunction	Type 1 (Figure 1, A): Localized low FAF signal at the fovea surrounded by a homogeneous background, with/without perifoveal foci of high or low FAF signal Type 2 (Figure 1, B): Localized low FAF signal at the macula surrounded by a heterogeneous background, and widespread foci of high or low FAF signal extending anterior to the vascular arcades Type 3 (Figure 1, C): Multiple areas of low FAF signal at the posterior pole with a heterogeneous background, with/without foci of high or low FAF signal

ERG = electroretinography; FAF = fundus autofluorescence.

### Best-Corrected Visual Acuity

Each patient underwent a best-corrected visual acuity (BCVA) assessment using the Early Treatment Diabetic Retinopathy Study chart at a testing distance of 4 m, 2 m, or 1 m depending on the patient's vision. The Early Treatment Diabetic Retinopathy Study chart was back-illuminated with a luminance of 100-125 cd/m^2^. If prescribed, the patient's distance refraction was corrected for using spectacles or contact lenses. The test was completed for each eye monocularly. A different chart was used for the right eye and left eye, such that the letters on the chart were varied for each assessment. Best-corrected Snellen visual acuity was converted to equivalent value of logarithm of minimal angle of resolution (logMAR) unit, and visual acuity reduction was calculated as the difference between logMAR visual acuity at baseline and at follow-up.

### Electroretinography

Patients were characterized into 3 electroretinography (ERG) groups, as previously described; Group 1 had dysfunction confined to the macula; Group 2 had macular and generalized cone system dysfunction; and Group 3 had macular and generalized both cone and rod system dysfunction.^3^ The ERG grouping is summarized in Table 1. ERG testing was not conducted as part of the study but was available from previous clinical evaluation at the time of baseline FAF assessment.

### Fundus Autofluorescence Imaging

FAF (short-wavelength) imaging was obtained using the Spectralis confocal scanning laser ophthalmoscope (Heidelberg Engineering, Heidelberg, Germany; excitation light, 488 nm; barrier filter, 500 nm; field of view, 30° × 30°), after pupil dilation using 1 drop each of tropicamide 1% and phenylephrine 2.5%, and with automated real-time tracking mode on. Laser power was set at 100% (conventional) and total sensitivity was adjusted for an optimal image exposure (“freely adjusted”) over an imaging duration of ~30 seconds.^38^

### FAF Qualitative Image Analysis

**Figure 1:**
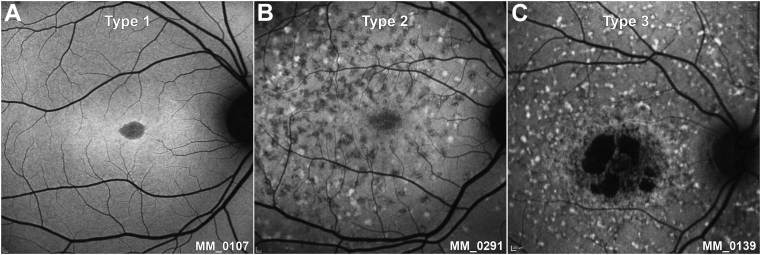
Fundus autofluorescence (FAF) types. (A) Type 1, localized low FAF signal at the fovea surrounded by a homogeneous background. (B) Type 2, localized low FAF signal at the macula surrounded by a heterogeneous background, and widespread foci of high or low FAF signal extending anterior to the vascular arcades. (C) Type 3, multiple areas of low FAF signal at the posterior pole with a heterogeneous background, with foci of high and low FAF signal.

Baseline and follow-up images (n = 360) were available and qualitatively graded for both eyes of all patients by 1 observer (M.G.). Patients were classified into 1 of 3 types of FAF pattern, as previously described.^9^^,^^39^ The 3 subtypes were as follows: type 1, localized low FAF signal at the fovea surrounded by a homogeneous background, with or without perifoveal foci of high or low FAF signal (Figure 1, A); type 2, localized low FAF signal at the macula surrounded by a heterogeneous background, and widespread foci of high or low FAF signal extending anterior to the vascular arcades (Figure 1, B); and type 3, multiple areas of low FAF signal at the posterior pole with a heterogeneous background, with or without foci of high or low FAF signal (Figure 1, C). Images of each FAF type are presented in Figure 1, and Table 1 summarizes the FAF grading system. Observer 1 (M.G.) also qualitatively assessed all images as to whether they were fit for quantitative analysis. The rate of progression was calculated for all 3 groups and each type of FAF at baseline.

### Repeatability and Reliability Assessment

Fifty FAF images were randomly selected to assess the intraobserver repeatability and interobserver reliability of the method used for quantitative assessment. A number (1-334) was allocated to 334 images considered for quantitative analysis and 50 numbers were randomly selected using a random number generator script (Python Software Foundation, version 3.5). The area of decreased autofluorescence (DAF) was calculated in the corresponding pictures, in a masked fashion, by 2 observers, observer 1 (M.G.) and observer 2 (T.K.), to assess interobserver reliability. Observer 1 also reassessed the images in a masked fashion, at least 1 week apart, to evaluate intraobserver repeatability. Both observers had previous experience (2 years) in analyzing retinal images of inherited retinal diseases but they were naïve to the method used here and were trained before this study using 10 STGD1 FAF images that were not included in this study.

### FAF Quantitative Image Analysis

The Heidelberg Spectralis Region Finder tool was used for semiautomated quantitative analysis of FAF images. Based on the extent of darkness, the observers defined areas of DAF qualitatively as being either definitely or questionably decreased as previously described.^40^^,^^41^ The reference points for determining the level of darkness of the lesions were the blood vessels and the optic disc as the absolute level of darkness at one end of the scale and the retinal background AF at the other end. Areas in which DAF was close (>90% of darkness) to the level of vessels and the optic disc were defined as definitely decreased AF (DDAF), and the areas of DAF between 50-90% of darkness, were defined as questionably decreased AF (QDAF).^38^ The level of darkness was subjectively estimated by the observers as in previous studies.^5^^,^^40^, ^41^, ^42^

**Figure 2:**
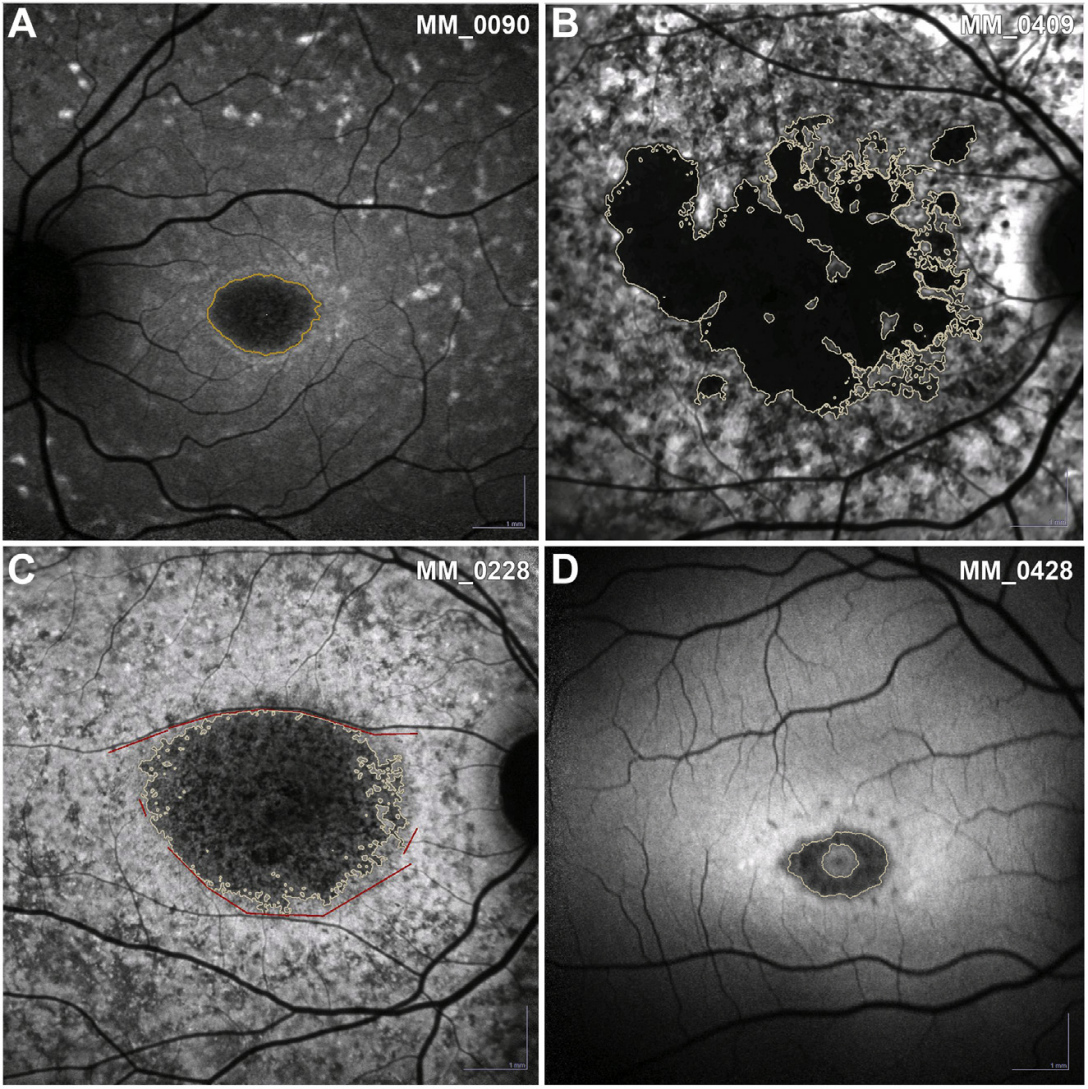
Method of decreased autofluorescence (DAF) quantitative analysis. (A) Outlined region of DAF after threshold adjustment. (B) Multifocal lesions, in which the sum of all areas of DAF was calculated. (C) Manual line and freehand constraints were used to distinguish lesion boundaries and to exclude vascular structures. (D) Areas of foveal sparing were delineated with freehand constraints after consulting the infrared images and the ring-shaped DAF was evaluated.

The observers selected seed points within candidate areas of atrophy by clicking on the darkest areas inside the lesion. The software considers all the adjacent pixels with a signal intensity equal to and below the signal intensity of the seed point and outlines the region. Observers make adjustments by changing the threshold of the region-growing algorithm to precisely outline the region (Figure 2, A). For multifocal lesions, the sum of all areas of DAF was calculated (Figure 2, B). In this study, we have used previously established conventions: 1) the minimum lesion size considered as an area of DAF was defined to have a diameter >125 μm and/or a lesion area of 0.012 mm^2^; 2) shadow correction was applied when the FAF images were unevenly or inadequately illuminated; 3) manual line, circles, contours, or free-hand constraints were used as needed to distinguish lesion boundaries and exclude vascular structures (Figure 2, C); 4) peripapillary atrophy was excluded from area calculation; 5) in case of confluence of central and peripapillary atrophy, an approximately vertical line constraint had to be set at the narrowest part (“bridge”), with atrophy quantification including only atrophy temporal to the constraint; and 6) areas of foveal sparing were delineated with free-hand constraints after consulting infrared images and/or OCT obtained at the same visit (Figure 2, D).^5^^,^^35^^,^^40^, ^41^, ^42^, ^43^

The baseline measurement of DAF (QDAF plus DDAF) and the rate of progression (mm^2^/year) were evaluated for disease symmetry between eyes. The rate of progression was calculated for each individual group of patients (children, adults with childhood-onset, or adult-onset STGD1). Correlation with age of onset, disease duration, ERG group at baseline, baseline measurements, and rate of progression was also investigated.

### Statistical Methods

The statistical analysis was carried out using SPSS software (IBM Corp., Armonk, NY). Significance for all statistical tests was set at *P* < .05. The Shapiro-Wilk test was used to test for normality for all variables. Parametric and nonparametric tests were used as appropriate. Reliability and repeatability were calculated as the intraclass correlation coefficient based on a 2-way mixed-effects model, absolute agreement, and with Bland-Altman analysis. A threshold of intraclass correlation coefficient >0.90, with the lower limit of the 95% confidence interval >0.75, was used to designate a reliable method. The interocular symmetry was assessed with a paired *t* test for DAF area, at baseline and follow-up, as well as annual rates of change. The correlations between eyes were assessed with either the Spearman or Pearson correlation coefficient as appropriate. Subsequently, the strength of the correlations between the baseline measurement and annual rate of progression, baseline measurement and age at baseline, as well as the age at baseline and annual rate of progression were assessed. Strong, moderate, and weak absolute correlation was defined as *r* > 0.7, 0.7 > *r* > 0.5, and *r* < 0.5, respectively.

## Results

### Demographics

Ninety patients with STGD1 were ascertained. Fifty-four patients were female (60%). Fifty-six were children at baseline FAF imaging (n = 56; age range, 6.5-16.1 years; mean age ± SD, 11.03 ± 2.96 years; mean follow-up ± SD, 3.5 ± 1.8 years) and 34 were adults (n = 34; age range, 17.8-66.2; mean age ± SD, 30.1 ± 10.46 years). Fifteen adults had childhood-onset disease (onset <17 years of age, n = 15; age range, 17.8-64.8; mean age, 26.3 years; mean follow-up ± SD, 3.4 ± 2.2 years), and the rest had adult-onset (onset ≥17 years of age, n = 19; age range, 20.2-48.4; mean age, 33.0 years; mean follow-up ± SD, 5.4 ± 3.5 years). The age of onset was documented in all subjects and was 9.6 ± 3.4 years for childhood-onset (n = 71) and 28.3 ± 7.8 years for adult-onset STGD1 (n = 19); with the presenting symptom in all patients being increased difficulty in seeing.

### Genetics

In documented childhood-onset disease (n = 71), ≥2 likely disease-causing variants were identified in 69 patients; with 4 patients having 3 variants and 2 siblings having 4 variants. For 2 patients, only 1 likely disease-causing variant was identified. In total, 148 variants were identified in the childhood-onset cohort, of which 95 were missense (64.2%), 25 splice-site alterations (16.9%), 10 nonsense (6.8%), 6 in-frame insertions (4.1%), 7 in-frame deletions (4.7%), and 5 disease-associated intronic variants (3.3%). The genetic findings of 46 of our childhood-onset patients have been previously reported.^36^ The genetic data are summarized in Supplemental Table (available at AJO.com).

The variant c.5882G>A, p.(Gly1961Glu), was the most commonly detected variant in both childhood and adult-onset groups. Nine alleles were identified (9/142, 6.3%) in childhood-onset and 10 alleles in adult-onset disease (10/38, 26.3%). All patients were classified based on the genotype group classification described in Table 1. The most common classification for childhood-onset was B (n = 31/71, 43.7%) and for adult-onset was C (n = 11/19, 57.9%). By definition B, with the presence of a null variant, suggests a more severe genetic background. At least 1 null variant (groups A and B) was identified in 38 of 71 patients with childhood-onset disease (53.5%) compared with 7 of 19 patients with adult-onset disease (36.8%).

### BCVA

BCVA was available for all patients both at baseline and follow-up. BCVA at baseline was similar between eyes (*P* = .859, *t* = 0.178, *df* = 89), with a mean ± SD of 0.68 ± 0.34 and 0.68 ± 0.32 logMAR for right and left eyes, respectively. BCVA at follow-up was also similar between eyes (*P* = .870, *t* = −0.164, *df* = 89), with a mean ± SD of 0.86 ± 0.32 and 0.87 ± 0.25 logMAR for the right and left eyes, respectively. The mean BCVA change (±SD) during follow-up was 0.18 ± 0.20 and 0.19 ± 0.19 logMAR for the right and left eyes, respectively. This mean change corresponds to a mean loss of 9 Early Treatment Diabetic Retinopathy Study letters over 4.2 years, and an average Snellen BCVA reduction from 20/90 (6/27) at baseline, to 20/145 (6/44) at follow-up. For 61 subjects (49 children and 12 adults), the baseline BCVA used in the analysis herein coincided with the time of disease diagnosis.

### ERG Group

The ERG group was available for 86 patients, 67 with childhood-onset and 19 with adult-onset disease. ERG was performed as part of their clinical investigation at or near the time of diagnosis. All patients had the same ERG group in both eyes. For childhood-onset disease, 39 (58.2%), 9 (13.4%), and 19 (28.4%) patients had ERG groups 1, 2, and 3, respectively. For adult-onset disease, 16 (84.2%), 1 (5.3%), and 1 (5.3%) patient(s) had ERG groups 1, 2, and 3, respectively. One adult had a pattern ERG within normal limits at the time of diagnosis (MM_0425, at 26 years of age, presented with subtle perifoveal changes and a BCVA of 0.18 and 0.0 logMAR). The childhood-onset cohort had a statistically significant greater number of patients in ERG groups 2 and 3 compared with the adult-onset (*P* = .0156, χ^2^ = 5.84). ERG groups 2 and 3, describe a more extensive/severe retinal dysfunction.

### FAF Qualitative Grading

FAF images (n = 360, baseline and follow-up for both eyes) were qualitatively graded into 1 of 3 AF types (Table 1, Figure 1). At baseline, 40 (71.4%) children had type 1 AF pattern and 12 (21.4%) had type 2 AF pattern. No children had type 3 AF, and 4 (7.1%) were indeterminate (described below). Sixteen (28.6%) children progressed to a more severe type during follow-up: 2 from indeterminate to type 1 and 1 from indeterminate to type 2; 11 from type 1 to type 2; and 2 from type 2 to type 3. The mean follow-up was 4.3 and 3.2 years for children with and without a change in type, respectively.

**Figure 3:**
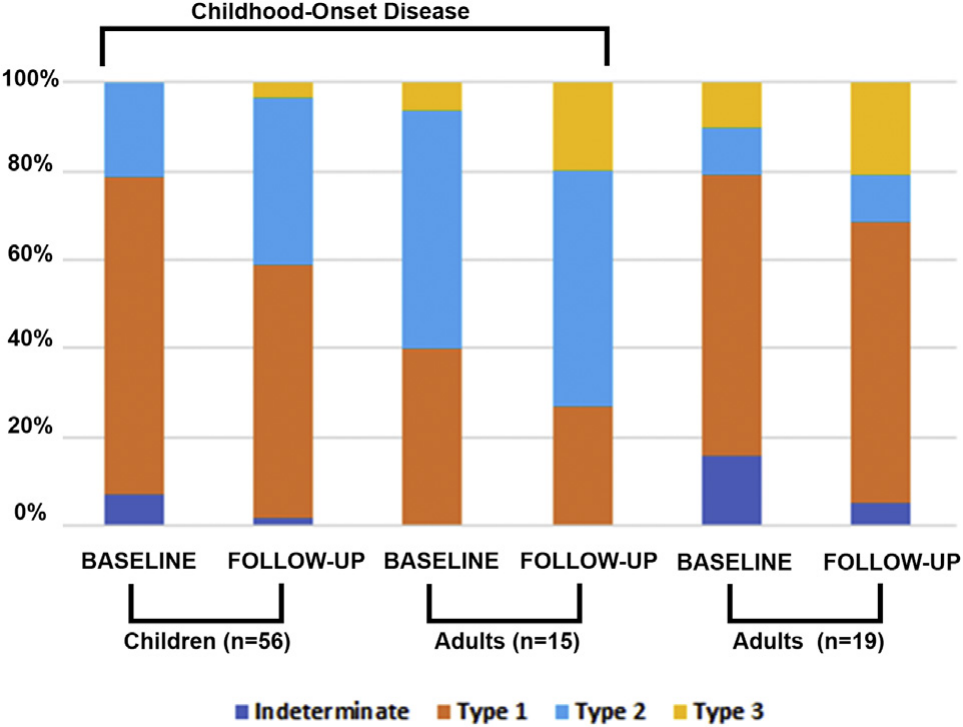
Categorization of fundus autofluorescence (FAF) by type and presentation. Colored bar charts depict the percentage of each type of FAF at baseline and follow-up for all 3 patient groups. There is a trend for childhood-onset disease over time (from baseline to follow-up and from children to adults) of a declining percentage in type 1 and an increasing percentage in type 2 and 3 (suggesting a worsening FAF phenotype over time).

At baseline, of the 15 adults with childhood-onset disease, 6 (40.0%) had type 1, 8 (53.3%) had type 2, and 1 (6.7%) had type 3. Four (26.6%) progressed to a more severe type during follow-up: 2 from type 1 to type 2, and 2 from type 2 to type 3. Of the 19 adult-onset patients, 12 (63.2%) had type 1, 2 (10.5%) had type 2, and 2 (10.5%) had type 3. Three were indeterminate (described below). Six (31.6%) progressed to a more severe type during follow-up: 2 from indeterminate to type 1; 2 from type 1 to type 2; and 2 from type 2 to type 3. The mean follow-up was 8.5 and 4.0 years for patients with and without a change in type, respectively. The results of qualitative analysis are summarized in Table 2 and Figure 3.

**Table 2:** Fundus Autofluorescence Type and Rate of Progression

Type, n (%)	Children, n = 56		Adults with Childhood-Onset, n = 15		Adults with Adult-Onset, n = 19	
	Baseline	Follow-Up	Baseline	Follow-Up	Baseline	Follow-Up
Indeterminate	4 (7)	1 (2)	0	0	3 (16)	1 (5)
Type 1	40 (71)	31 (55)	6 (40)	4 (27)	12 (63)	12 (63)
Type 2	12 (21)	22 (39)	8 (53)	8 (53)	2 (11)	2 (11)
Type 3	0	2 (4)	1 (7)	3 (20)	2 (11)	4 (21)

Rate of progression, mm^2^/year^a^						
Indeterminate	0.94		NA		0.13	
Type 1	0.61		0.46		0.41	
Type 2	0.74		0.95		0.63	
Type 3	NA		0.30		0.77	

a Based on baseline fundus autofluorescence type.

**Figure 4:**
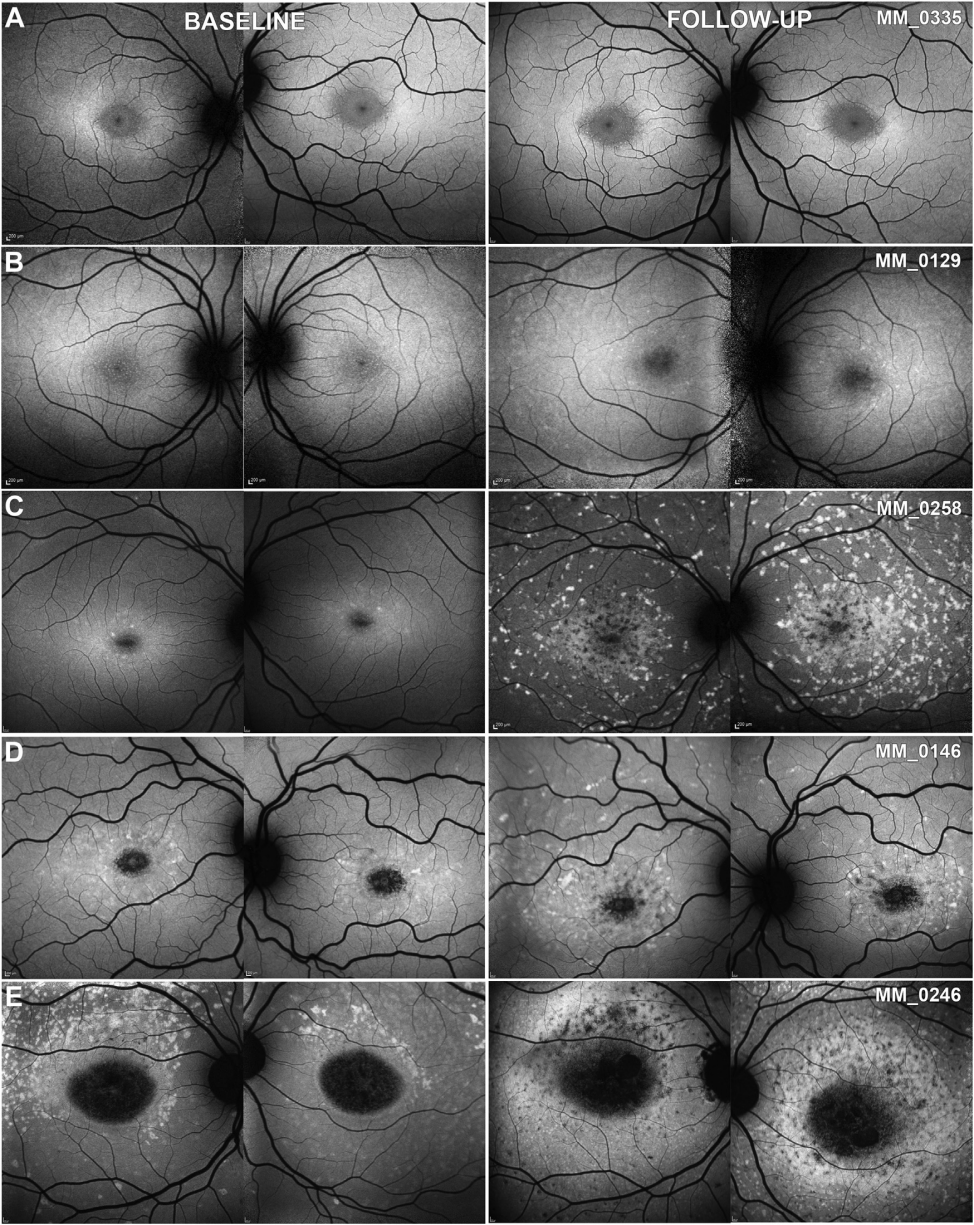
Quantitative analysis of fundus autofluorescence (FAF). FAF images from both eyes of 5 children at baseline and follow-up. All patients have the same FAF type between eyes. (A) Indeterminate FAF at both time points. (B) Progression from indeterminate to type 1. (C) Progression from indeterminate to type 2. (D) Progression from type 1 to type 2. (E) Progression from type 2 to type 3. All images are to scale.

**Figure 5:**
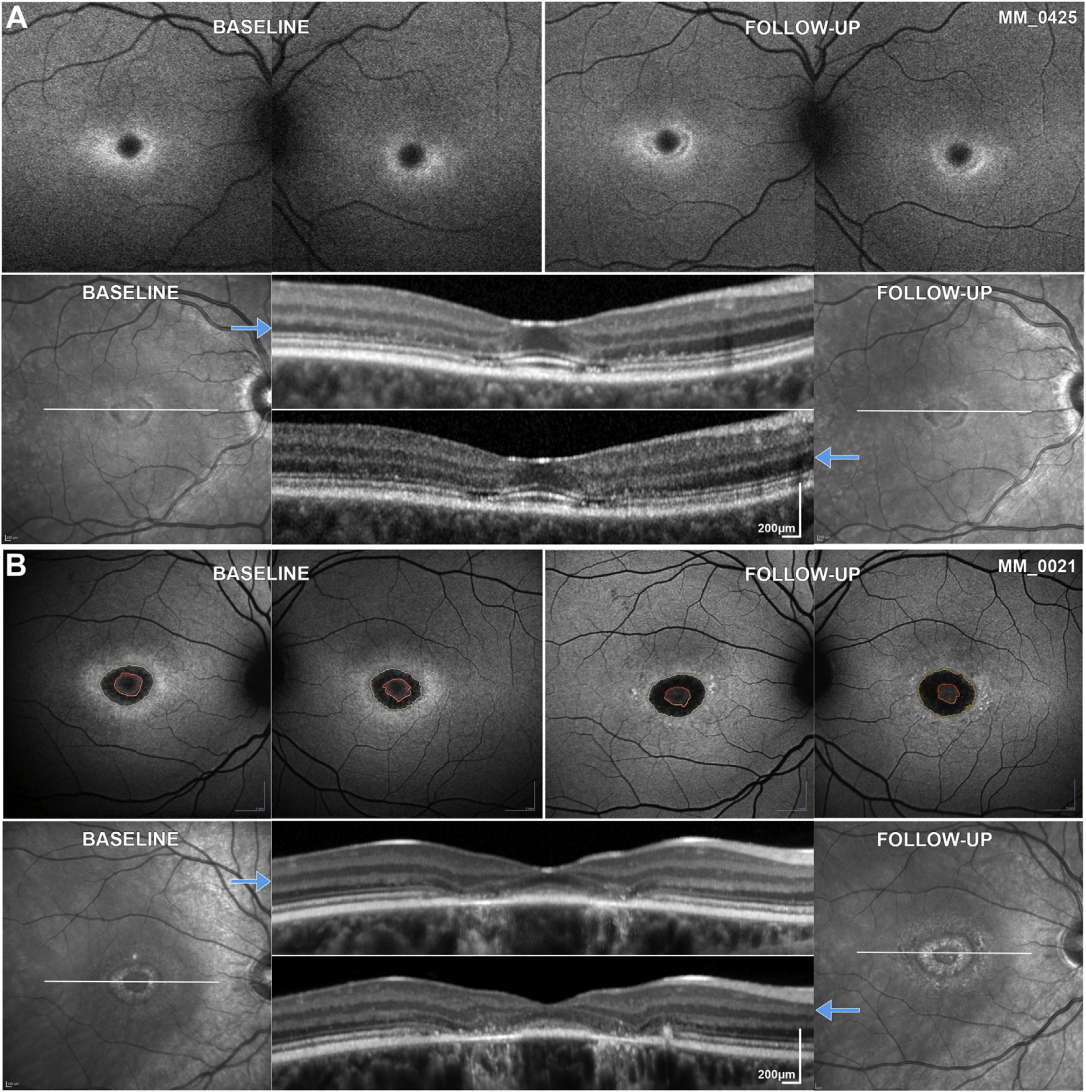
Intrafamilial variability. (A and B) First line: fundus autofluorescence (FAF) images at baseline and follow-up of both eyes. Second line: infrared image of the right eye at baseline and follow-up; the white horizontal lines mark the corresponding location of the optical coherence tomography (OCT) B-scan indicated by the blue arrows. (A) MM_0425 and (B) MM_0021 are siblings with ages of disease onset at 26 and 14 years of age, respectively. The younger sibling (B) was diagnosed earlier. Electroretinography at baseline was within normal limits for the older sister (A) and electroretinography group 1 for the younger brother (B). The patients have a mild genotype, harbouring the missense variants p.Gly1961Glu and p.Ala1598Asp. Both siblings had foveal sparing and the rate of progression was among the lowest in the study (0.08 and 0.21 mm^2^/year, respectively). Both have perifoveal ellipsoid zone changes on OCT. FAF and OCT images are to scale within modalities. Foveal sparing is clearly visible on the infrared and OCT images. These images were used in outlining the normal decreased autofluorescence of the fovea and exclude the area [red delineated area on (B)] when quantifying the abnormal decreased autofluorescence.

Seven patients, 4 children (4/56, 7.1%) and 3 adults with adult-onset disease (3/19, 15.8%), all of whom were recruited at the time of initial diagnosis, had no obvious abnormalities or findings meeting the description of the FAF grading system. Five of them progressed to 1 of the 3 FAF types during follow-up (Figure 4, B and C). One child (MM_0335; Figure 4, A) and 1 adult (MM_0425) had minimal signs of progression over a follow-up period of 2.9 and 1.4 years, respectively, with a small perifoveal area of DAF only in MM_0425 (Figure 5, A). In contrast, OCT imaging revealed perifoveal disruption at baseline with minimal, if any, progression over time in both patients (Figure 5, A).

After qualitative grading, 26 images from 10 subjects were excluded from additional quantitative analysis because of absence of an area of DAF in 16 images (DAF = 0 mm^2^, 7 subjects, SM2-A); an area of DAF extending beyond FAF image borders in 6 images (2 subjects, SM2-C/D); and inadequate image quality for quantitative analysis in 4 images (1 subject, SM2-B). Examples of the excluded images are presented in the Supplemental Material (Supplemental Material 2). The calculated rate of progression for each type of FAF pattern at baseline in all 3 groups is shown in Table 2.

### Intraobserver and Interobserver Reliability

Fifty images were randomly selected after qualitative assessment and were quantitatively assessed in a masked fashion twice by observer 1 (M.G.) and once by observer 2 (T.K.) to evaluate intra- and interobserver reliability, respectively. Initially, all pictures were quantified and DAF classed as either DDAF or QDAF. The area measurements of DAF were similar for the 2 observers in most cases; however, for 21 (42%) images there were discrepancies as to whether there was QDAF, DDAF, or both, either between the observers or between repeat assessments by observer 1. Even though the definitions of QDAF and DDAF are not complex, the decision on whether an area has 50-90% or 90-100% DAF is often subjective and variable. We therefore decided to only compare measurements of DAF.

**Figure 6:**
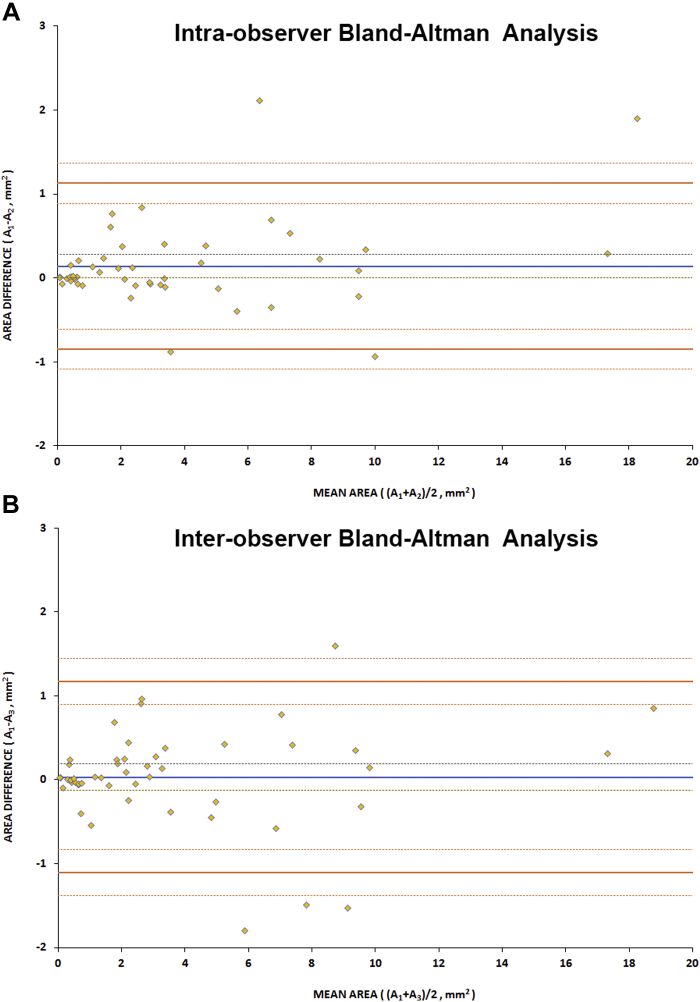
Bland-Altman reliability assessment for quantitative analysis of fundus autofluorescence. The blue line represents the mean difference and the dashed lines represent the upper and lower standard error. The orange lines represent the upper and lower limits of agreement, with the dashed lines around them their upper and lower confidence intervals. (A) Analysis of intraobserver agreement. (B) Analysis of interobserver agreement. Only 6 pairs of measurements are lying outside the limits of agreement. No bias was observed either for intra- or interobserver analysis. A_1_ = first area measurements for observer 1; A_2_ = second area measurements for observer 1; A_3_ = area measurements for observer 2.

The intraclass correlation coefficient was calculated as 0.995 and 0.987 for intra- and interobserver agreement, respectively, representing excellent agreement. Bland-Altman analysis was also performed and no bias was found for either intra- or interobserver measurements.^44^ Six outliers were identified (of the 100 total sets of measurements) and all represented cases with a mean area >4 mm^2^. For small lesions there was a clustering effect near the origin on Bland-Altman intra- and interobserver analysis graphs, representing high repeatability and reproducibility in their quantification. Reliability results are summarized in Table 3. Bland-Altman plots are presented in Figure 6. The mean (±SD) absolute difference was 0.29 mm^2^ (±0.43 mm^2^) and 0.37 mm^2^ (±0.44 mm^2^) for observer 1 and between observers, respectively. Subsequent quantitative analysis in all 334 images was done by a single observer (M.G.) given the excellent intra- and interobserver repeatability agreement.

**Table 3:** Reliability Results of Fundus Autofluorescence Quantitative Analysis

Parameter	Intraobserver	Interobserver
ICC (95% CI)	0.995 (0.985 to 0.998)	0.987 (0.963 to 0.996)
Bland-Altman analysis, mm^2^		
Bias (95% CI)	0.14 (0.0 to 0.28)	0.03 (−0.13 to 0.19)
Upper LOA (95% CI)	1.13 (0.89 to 1.37)	1.17 (0.89 to 1.45)
Lower LOA (95% CI)	−0.85 (−1.09 to −0.61)	−1.11 (−1.38 to −0.83)

CI= confidence interval; ICC = intraclass correlation coefficient; LOA = limit of agreement.

### Interocular Symmetry

**Figure 7:**
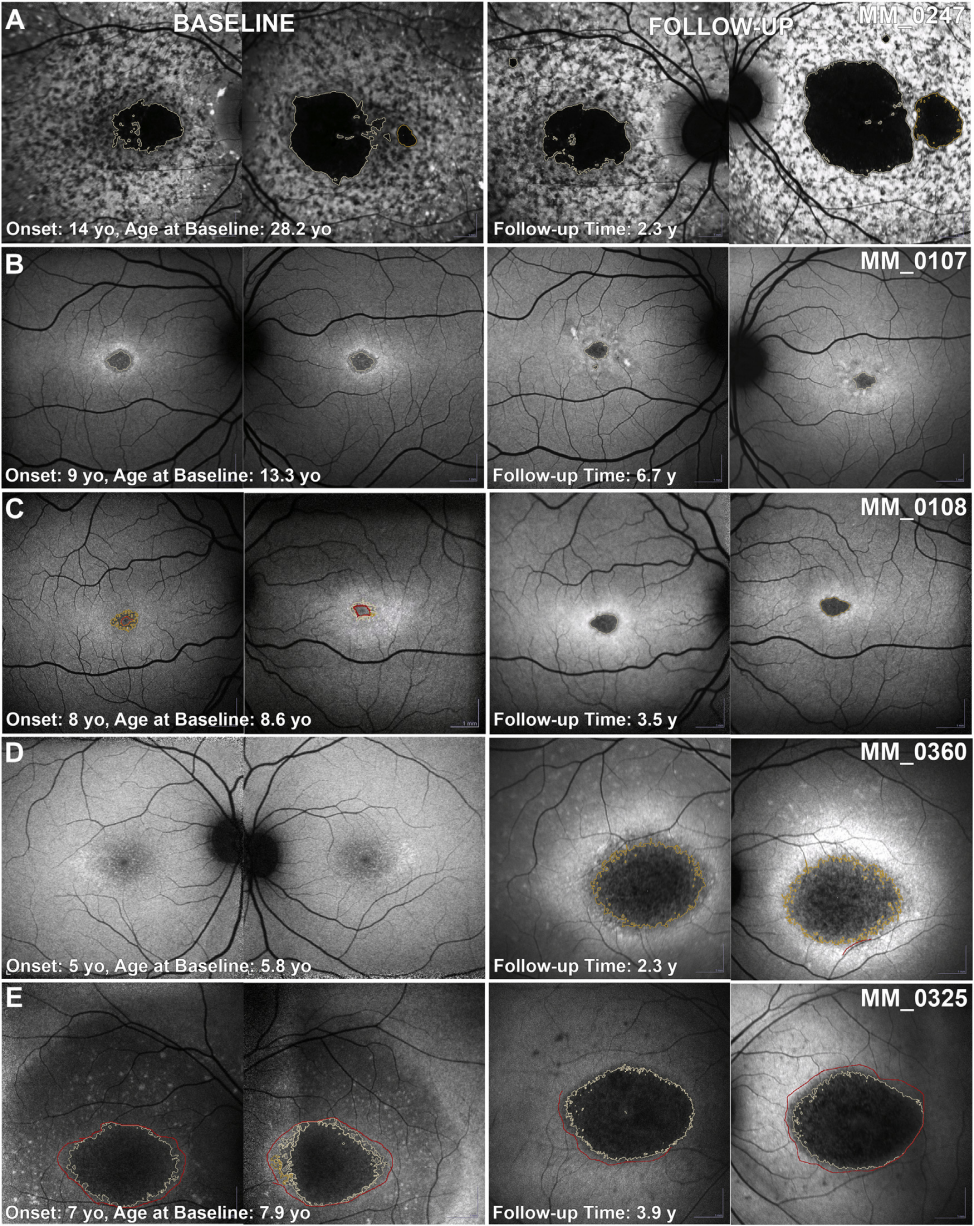
Fundus autofluorescence progression. Fundus autofluorescence images from both eyes of 5 patients with childhood-onset disease at baseline and follow-up. Patient (A) had asymmetric disease at baseline and follow-up, and an asymmetric rate of progression. (B-E) All patients have symmetric disease between eyes. (B and C) and (D and E) are siblings and have a similar disease course. Patients (B) and (C) have a minimal rate of progression of 0.01 and 0.06 mm^2^/year, respectively. Patients (D) and (E) have a rapidly progressive disease, and are homozygous for the p.Gly72Arg variant. All images are to scale. y = years; yo = years old.

Interocular symmetry was evaluated both for qualitative and quantitative FAF assessment. FAF type was the same bilaterally in all patients both at baseline and follow-up (Figure 4, Figure 7, B through E), with the exception of 2 patients with different types between eyes; 1 at baseline and 1 at follow-up visit (Figure 7, A). Both patients were adults, with advanced disease bilaterally (MM_0242 and MM_0247). The only patient with a different type at baseline had the same type in both eyes at follow-up. Examples of AF type disease symmetry and progression are presented in Figure 4, Figure 7.

Eighty-one pairs of eyes were further quantitatively analyzed at baseline (7 pairs were excluded because of a lack of DAF, 1 because of poor image quality and 1 because of extensive DAF area beyond the image borders; Supplemental Material 2). The area of DAF (QDAF plus DDAF) was similar between eyes (*P* = .658, *t* = −0.444, *df* = 80), with mean absolute difference (±SD) of 0.33 mm^2^ (±1.06 mm^2^). Only 3 subjects (3.7%) had interocular DAF difference >1 mm^2^ and were identified as outliers (MM_0065, MM_0161, and MM_0247, with interocular differences of 5.90, 5.22, and 5.88 mm^2^, respectively), with obvious asymmetry between the eyes (Figure 7, A).

At the follow-up visit, 86 pairs of eyes were analyzed (1 pair was excluded because of a lack of DAF, 2 because of poor image quality, and 1 because of extensive DAF area beyond the image borders). The mean absolute difference (±SD) between the right and left eyes was 0.57 mm^2^ (±1.40 mm^2^). Even though the area of DAF was similar between the eyes (*P* = .647, *t* = 0.459, *df* = 85), 2 patients were noted to have asymmetric DAF (MM_0019 and MM_0409, with interocular differences of 1.66 and 3.31 mm^2^, respectively).

**Figure 8:**
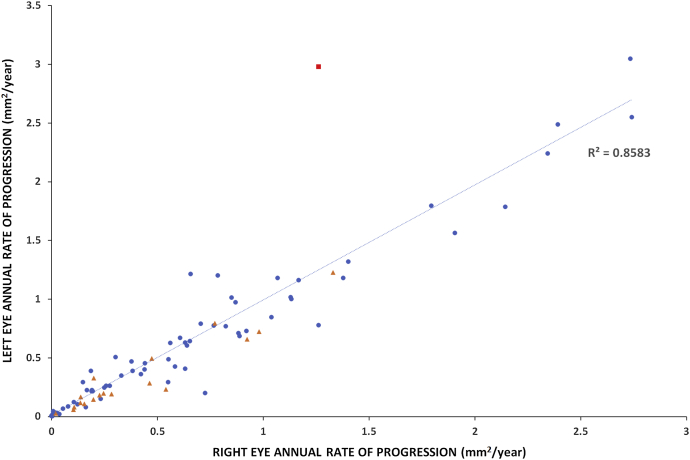
Progression symmetry. Scatter plot presenting the rate of progression for each pair of eyes, with line of best fit. The blue dots correspond to childhood-onset patients and the orange triangles to adult-onset patients. The rate of progression was highly variable from 0-3 mm^2^/year, with almost all patients having symmetric progression. Patient MM_0247 (Figure 7, A) has a different rate of progression among eyes and was an outlier (red square on the graph).

Finally, the rate of progression was evaluated for both eyes of 86 patients. The mean absolute difference between eyes (range [±SD]) was 0.13 mm^2^/year (0-1.71 [±0.22] mm^2^/year). Figure 8 presents the rate of progression for each pair of eyes, with the line of best fit. Patient MM_0247 had a different rate of progression between eyes and was an outlier (Figure 7, A, and the red square on graph Figure 8). All patients (n = 5, 5.6%) with asymmetry at baseline, and/or follow-up, and/or rate of progression were adults with advanced disease and who had a mean disease duration of 18.8 years (range, 5-50 years; median, 14 years). All children had symmetric disease without a significant difference between eyes at baseline (*P* = .909, *t* = −0.114, *df* = 52) and in rate of progression (*P* = .191, *t* = 1.33, *df* = 52; Figure 7, B through E).

### Rate of Progression

**Figure 9:**
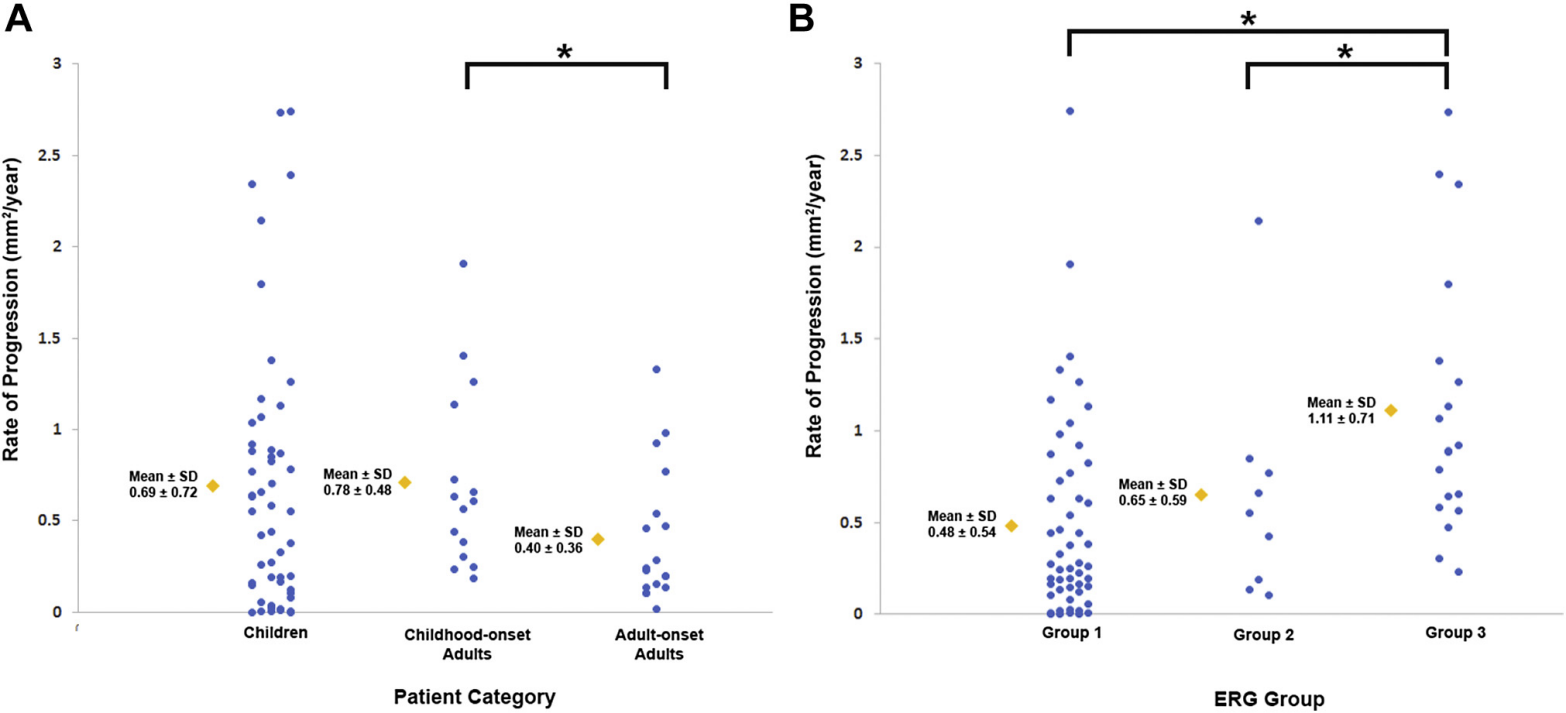
Stacked scatter plots for rate of progression for patient category and electroretinography group. Blue circles represent the progression rate for patients in the corresponding category or group and are displaced horizontally to aid visualization. Yellow diamonds represent the mean value. Asterisks mark statistically significant differences (*P* < .05). (A) Rate of progression for each group of patients: the rate of progression was highest for adults with childhood-onset disease, followed by children with childhood-onset disease. In all 3 categories the rate was highly variable. (B) Rate of progression for each electroretinography group: the rate of progression was statistically significantly higher for group 3. In all 3 groups the rate was again highly variable.

Data from the right eyes of all subjects were used for this analysis, after investigating disease symmetry and in order to avoid any clustering effect. The mean (±SD) DAF area at baseline was 2.59 (±5.58) mm^2^ for children, 3.74 (±4.57) for adults with childhood-onset, and 2.15 (±2.12) for adults with adult-onset disease. The rate of progression was evaluated for 86 patients (1 patient was excluded because of a lack of DAF, 1 because of poor image quality, and 2 because of extensive DAF area beyond the image borders). The mean annual rate (±SD) of progression was calculated for each group individually and was 0.69 (±0.72) mm^2^/year for children (n = 53), 0.71 ± 0.48 mm^2^/year for adults with childhood-onset (n = 15), and 0.40 ± 0.36 mm^2^/year for adults with adult-onset disease (n = 18). The rate of progression was significantly lower for adult-onset disease compared with adults with childhood-onset disease (*P* = .022, U = 72, n_2_ = 18, and n_3_ = 15). The rate of progression was similar for children and adults with childhood-onset disease (*P* = .315, U = 330, n_1_ = 53, and n_2_ = 15). As shown in Figure 7, Figure 9, the rate of progression was highly variable within the 3 cohorts.

The rate of progression is weakly, but significantly, negatively associated with age of onset (*P* = .05, *r* = −0.214, n = 86), and is in keeping with a slower rate of progression with an older age of onset. Disease duration (age of onset to last follow-up) was not associated with progression rate (*P* = .56, *r* = −0.065, n = 86). The rate of progression showed a statistically significant moderate positive correlation with the size of the area of DAF at baseline (*P* < .001, *r* = 0.46, n = 86), indicating a faster rate of progression with larger baseline lesion size.

Rate of progression and ERG group was available for 81 patients. The mean rate of progression ±SD was 0.48 ± 0.54 mm^2^/year for ERG group 1 (n_1_ = 53), 0.65 ± 0.59 mm^2^/year for group 2 (n_2_ = 9), and 1.11 ± 0.71 mm^2^/year for group 3 (n_3_ = 19). Group 3 had a statistically significant faster rate of progression compared with both group 1 (*P* = .00012, U = 202, n_1_ = 53, and n_3_ = 19) and group 2 (*P* = .037, U = 43, n_2_ = 9, and n_3_ = 19). There was no statistically significant difference between group 1 and group 2 (*P* = .313, U = 188, n_1_ = 53, and n_2_ = 9). Figure 9, B presents the rates of progression between the different ERG groups.

### Genotype–Phenotype Correlations

Variants that were identified in more than one patient with childhood-onset disease and in one or more patients in the homozygous state are described here.

#### p.(Gly72Arg)

This variant was identified in 3 siblings in the homozygous state. All 3 had childhood-onset disease (age range, 5.8-7.9 years). Visual acuity ranged from 1.1 logMAR for the first child to be diagnosed to 0.26 logMAR for the last child to be diagnosed at 5.8 years of age. BCVA was 0.96-1.2 logMAR at the follow-up visit (2.3-3.8 years of follow-up). All 3 had a homogenous background FAF at baseline. They had 3 of the highest progression rates in the entire cohort (0.92, 2.73, and 2.39 mm^2^/year; Figure 7, D and E). The young child had no area of quantifiable DAF at baseline (Figure 7, D). All 3 children had ERG group 3. The predicted effect of this variant has been previously described by Garces and associates as severe, and they described 2 compound heterozygous siblings [p.(Gly72Arg)/p.(Gly1961Glu)] with a milder phenotype than the 3 cases herein.^45^

#### p.(Leu2027Phe)

This missense variant was previously reported to cause a mild phenotype, a smaller area of central atrophy, and a slow rate of progression.^9^^,^^39^^,^^46^^,^^47^ In our cohort a broad range of clinical phenotypes were identified associated with this substitution. The 1 patient homozygous for this variant had childhood-onset disease (13 years of age) and a progression rate of 1.4 mm^2^/year, which is toward the high end of the range observed in this study. Four other patients harbored the variant in the heterozygous state with a null variant (genotype group B), all had childhood-onset disease (age of onset, 7-16 years) and a highly variable range of progression (0.13-1.91 mm^2^/year). Two of the patients were siblings (MM_0128 and MM_0129), diagnosed at 12 (younger sibling; Figure 4, B) and at 7 (older sibling) years of age, and showed minimal to slow rates of progression (0.123 and 0.55 mm^2^/year, respectively). The older sibling had a normal FAF at baseline and progressed the least (Figure 4, B). At final follow-up, both patients presented with type 1 FAF. Interestingly, there was discordance between the siblings, with the age of onset being 5 years later in the younger sibling and associated with better BCVA compared with the older sibling. Another patient harboring the variant in the heterozygous state with another missense variant [p.(Phe418Ser); genotype group C] had adult-onset disease (35 years of age) with a relatively high rate of progression (0.98 mm^2^/year) and visual acuity of 1.32 logMAR at last follow-up, 15 years after disease onset (Figure 2, B).

#### p.(Phe418Ser)

The only patient homozygous for this variant had childhood-onset disease (7 years of age) and a minimal progression rate of 0.039 mm^2^/year, which is one of the lowest observed in this study, with the patient being essentially stable over a follow-up time of 3.5 years. Despite the disease stability, BCVA was already severely reduced at baseline to 1.0/0.9 logMAR and 1.0/0.94 logMAR at follow-up. The other 3 patients have a group C genotype (missense variants only). One patient was heterozygous for the variant with p.(Leu2027Phe) and has been described above (Figure 2, B). One patient was heterozygous for variant p.(Arg1108Cys) and presented with childhood-onset disease (onset at 7 years of age) and was stable over 2.9 years of follow-up (rate of progression, 0.005 mm^2^/year). The final patient was heterozygous for the variant p.(Gly863Ala) and presented with early adult-onset disease (onset at 18 years of age) and foveal sparing (Figure 2, D), with a low rate of progression (0.15 mm^2^/year) over a follow-up time of 2.9 years. The above findings suggest that p.(Phe418Ser) is a mild allele and is associated with milder phenotypes of often childhood-onset disease.

#### c.6729+5_19delGTTGGCCCTGGGGCA

Three children were homozygous for this splice site alteration. Although the 3 patients were attributed to 3 different pedigrees, they all originated from the same Indian-Pakistani community and it is possible that they are members of 1 extended family or the result of a founder effect. The age of disease onset was between 4 and 6 years of age. At follow-up visit, BCVA was 0.9-1.10 logMAR (8.4-12.9 years of age). One patient (MM_0426) was the only subject in this study with poor image quality for reliable quantitative assessment (Supplemental Material 2,B). For the other 2 patients, the progression rate was toward the high end of the range (0.85 and 2.34 mm^2^/year). All 3 patients both at baseline and follow-up had a homogenous FAF background. This splice site alteration appears to be a severe allele and results in a rapidly progressive early childhood-onset disease.

### Intrafamilial Variability

In our study we had 8 pairs and 1 triplet of siblings. There was minimal intrafamilial variability observed. The triplet of siblings (MM_0325, MM_0326, and MM_0360) homozygous for p.(Gly72Arg), and the 1 pair of siblings (MM_0128, MM_0129) heterozygous for p.(Leu2027Phe) are described above in the genotype–phenotype correlations. For the remaining 7 pairs, the age of onset differed by <2 years for 5 of them. ERG testing was available for both siblings in 5 pairs, and from those, 4 had the same ERG group. In the remaining siblings, ERG data were available only for 1 patient (MM_0020, group 3), with a rate of progression of 0.89 mm^2^/year. A comparable rate of 0.71 mm^2^/year was observed in the younger sibling. Figure 7, B and C presents a pair of siblings following the same course with minimal progression.

In 1 pair (MM_0021-MM_0425; Figure 5), the age of onset was 14 and 26 years of age for the younger and older sibling, respectively, with the younger one being diagnosed earlier than the older one. ERG at baseline was within normal limits for the elder sister (MM_0425; Figure 5, A) and group 1 for the younger brother (MM_0021; Figure 5, B). The patients had a group C genotype harboring the missense variants p.(Gly1961Glu) and p.(Ala1598Asp). Both siblings had foveal sparing and the rate of progression was among the lowest in the study (0.08 and 0.21 mm^2^/year). The elder sister had a normal-appearing FAF at baseline, with decreased BCVA in the right eye (0.18 logMAR) at presentation and perifoveal ellipsoid zone (EZ) changes on OCT (Figures 5, A). A similar presentation was observed in patient MM_0335 (Supplemental Material 2,A), the sibling of MM_0336, and over the follow-up period no DAF was observed on FAF. The remaining pairs of siblings had the same FAF type at baseline and followed a similar course of disease over time.

## Discussion

This study reports the findings of the largest prospective cohort study of children with molecularly confirmed STGD1, including qualitative and quantitative FAF assessment, genetic background, and clinical information. We evaluated the reliability and repeatability of quantitative DAF evaluation and investigated disease symmetry, genotype correlations, and intrafamilial variability.

In the current literature the reported rate of progression is highly variable, ranging from 0.28-1.58 mm^2^/year.^9^^,^^29^^,^^31^^,^^34^^,^^41^^,^^48^ There are multiple inherent limitations of many of the studies that have reported these rates of progression, including retrospective nature, the patients not being molecularly confirmed, small cohorts, a heterogeneous groups of patients in terms of both age and disease onset (3 distinct phenotypic [and genetic] groups often analyzed together, namely childhood-onset, adult-onset, and late-onset), variable follow-up duration, and lack of reliability/repeatability assessment of the method used. In this prospective study we investigated the rate of progression by also considering the principal aforementioned limitations of the previous studies, including analyzing patients in 3 distinct molecularly confirmed and clinically well-characterized prospective cohorts and by assessing the reliability and repeatability of our method. After accounting for the limitations of previous studies, we identify a different rate of progression for each of our groups.

The semiautomated method used in the study had excellent reliability and repeatability in evaluating areas of DAF. The distinction between questionably and definitely decreased autofluorescence was difficult to establish in a masked fashion, as has been reported previously.^38^^,^^40^ The rate of progression was higher for childhood-onset disease compared with adult-onset disease and was faster for adults with childhood-onset disease compared with children with childhood-onset disease (Figure 9). These data suggest that patients with childhood-onset disease maintain a higher rate of progression throughout the course of the disease, that is, into adulthood. This higher rate compared with the rate observed during childhood can also be attributed to the largest baseline size of DAF area in the entire study, which has been shown to correlate with the rate of progression both in our study and previously, with a greater rate of progression associated with larger DAF area at baseline.^9^^,^^41^ Despite the mean rate of progression being notably higher in children compared with adults with adult-onset (0.68 compared with 0.40 mm^2^/year), this did not reach statistical significance; this may likely be because of the high variation in the children group (SD, 0.72 mm^2^/year)—with childhood-onset disease being highly heterogeneous in terms of progression rates (Figure 7, Figure 9).

A recent study describes a strong correlation between outer retinal degeneration and choriocapillaris loss in STGD using *en face* OCT and OCT angiography, with an EZ loss 1.6-fold greater than RPE atrophy.^49^ In agreement with the aforementioned study, it is noteworthy that the ratio of EZ loss reported in our recent OCT study^36^ (1.20 mm^2^/year, cohort of children with STGD1, n = 46) to the rate of DAF change calculated in the current study herein (0.69 mm^2^/year) is comparable (1.7). These observations also support the theory that photoreceptor degeneration precedes RPE loss in STGD1, or that functional RPE loss precedes the structural loss of RPE leading to photoreceptor loss before structural damage becomes apparent on FAF—this is in direct contrast to prevailing pathogenesis descriptions alluding to RPE loss preceding photoreceptor degeneration and may thereby necessitate a paradigm shift.^15^

Khan and associates investigated the early patterns of macular degeneration in childhood-onset STGD1 and observed parafoveal changes detected earlier with OCT than FAF; an observation we have also seen both in adults (Figure 5, A) and children in this study.^37^ OCT is more sensitive in identifying early changes in disease compared with FAF (higher resolution and 3-dimensional). However, quantifying EZ area loss using OCT can be significantly more laborious and time consuming,^50^ the field of view is smaller compared with FAF, and patients cannot be monitored if the atrophy extends beyond the border of the scan,^36^ a limitation that may be overcome with swept source OCT.^51^ Of note, the FAF acquisition rate and analyzable data were greater in the current study compared with our previous OCT study in a pediatric population, with 51 of 53 children being analyzed.^36^

More advanced recent variant analysis, such as polymerase chain reaction enrichment–based next generation sequencing, has resulted in a higher variant detection rate, including identifying the often “missing” second *ABCA4* allele, and thereby allowing more informed genotype–phenotype correlations to be investigated. It has been challenging in STGD1 to establish comprehensive genotype–phenotype correlations because of the highly variable phenotype and the vast allelic heterogeneity of *ABCA4*.^1^^,^^3^^,^^9^^,^^52^^,^^53^ The genotype–phenotype correlations we draw herein are important for patient counselling and prognosis determination. Intrafamilial variability was minimal, with a similar age of onset and disease course between siblings. Interestingly, the 2 pairs of siblings (MM_0021/MM_0425 and MM_0128/MM_0129) showing a greater intrafamilial variation had milder phenotypes (Figure 5).

A high degree of disease symmetry both at baseline and in terms of progression was observed between eyes, with few exceptions, and limited to adults with advanced disease (Figure 7, Figure 8). Previous studies have also described both structural symmetry on OCT and functional symmetry with retinal sensitivity testing (microperimetry and static perimetry) in children.^36^^,^^54^ Symmetry is important for any potential intervention, for which the fellow eye can serve as a control. Given the ongoing and upcoming clinical trials targeting STGD1, identification of robust endpoints is needed. BCVA was similar between eyes, and we observed a substantial reduction over a mean follow-up of 4.2 years. A previous study in a large cohort reported a minimal change over a follow-up period of 2 years.^55^ Plausible explanations for this difference include the higher proportion of childhood-onset patients in our study and the fact that the baseline BCVA often coincided with the time of disease diagnosis (especially in the children). In terms of prognosis, ERG groups with generalized retinal dysfunction (groups 2 and 3) were associated with a more rapid rate of DAF progression (Figure 9, B), in agreement with the retrospective studies in the literature.^3^^,^^28^^,^^52^ The more rapid deterioration of vision in our cohort and the greater proportion of patients with group 3 ERG in the childhood-onset cohort emphasizes the need for any potential “rescue” intervention to be applied early in the course of disease, and that children (and adults with childhood-onset disease) will likely help establish an efficacy signal more rapidly (compared with adult-onset disease) given their more rapid rate of progression.

In this study we have performed a qualitative and quantitative analysis of the area of DAF. Additional studies are needed to investigate the qualitative change of DAF in this population, as well as the correlation of the area of RPE loss using *en face* OCT with the area of DAF on FAF. The nonquantitative AF assessment (FAF pattern) can be more useful in the clinical assessment of individuals (together with ERG group). However, for patients progressing from one disease stage to another, this approach can be challenging when trying to assign them a grade (illustrated in Figure 4, B and C). In our cohort, we had a greater representation of patients with childhood-onset disease. Further investigations and comparisons between childhood-onset disease and a larger cohort of adult-onset disease will be valuable.

This is the largest prospective study characterizing FAF in a cohort of molecularly confirmed children with STGD1. The high intra- and interobserver agreement in DAF area quantification suggests that DAF can serve as a robust anatomic outcome measure in children. The data in this study are in keeping with childhood-onset disease being more severe than adult-onset disease. Rapid deterioration of vision in the first years after disease onset and the higher rates of progression were maintained into adulthood. Genetic background, ERG group, and intrafamilial presentation are of value in informing counselling of patients about prognosis.
