# Supplementary material for: Prospective Cohort Study of Childhood-Onset Stargardt Disease: Fundus Autofluorescence Imaging, Progression, Comparison with Adult-Onset Disease, and Disease Symmetry
Source: Am J Ophthalmol. 2020 Mar;211:159–75. doi: 10.1016/j.ajo.2019.11.008 (PMC7082771; doi:10.1016/j.ajo.2019.11.008)
Supplement: Supplemental Table [file mmc1.pdf]

Supplementary Table 1

Children at Baseline with Childhood-onset Disease

	Patient ID	Sex	Pedigree	Age of Disease Onset	ERG Group	Genotype Classification	Variants	
							cDNA-Change	Protein Effect
1	**MM_0020***	F	20660	7	3	B	c.4918C>T;c.4222T>C	p.Arg1640Trp;p.Trp1408Arg
							c.247_248insCAAA	p.Gln83ProfsTer17
2	**MM_0021***	M	31700	14	1	C	c.5882G>A	p.Gly1961Glu
							c.4793C>A	p.Ala1598Asp
3	**MM_0026***	F	20553	9	3	C	c.5549T>C	p.Leu1850Pro
							c.4469G>A	p.Cys1490Tyr
4	**MM_0034**	F	20096	14	1	C	c.5882G>A	p.Gly1961Glu
							c.1531C>T	p.Arg511Cys
							c.872C>T	p.Pro291Leu
							**c.5218_5219insCTG**	**In-frame insertion**
5	**MM_0035**	F	20096	14	1	C	c.5882G>A	p.Gly1961Glu
							c.1531C>T	p.Arg511Cys
							c.872C>T	p.Pro291Leu
							**c.5218_5219insCTG**	**In-frame insertion**
6	**MM_0058***	M	20002	14	1	B	c.5882G>A	p.Gly1961Glu
							c.454C>T	p.Arg152Ter
7	**MM_0070***	F	20892	11	1	C	c.5882G>A;c.3758C>T	p.Gly1961Glu;p.Thr1253Met
							c.3364G>A	p.Glu1122Lys
8	**MM_0090***	F	20935	13	1	B	c.3322C>T	p.Arg1108Cys
							c.3210_3211insGT	p.Ser1071CysfsTer14
9	**MM_0107***	F	19545	9	1	B	c.5161_5162delAC	p.Thr1721TfsTer65
							c.2588G>C	p.Gly863Ala
10	**MM_0108***	F	19545	8	1	B	c.5161_5162delAC	p.Thr1721TfsTer65
							c.2588G>C	p.Gly863Ala
11	**MM_0113***	F	19820	11	1	A	c.6817-2A>C	Splice site alteration
							c.1906C>T	p.Gln636Ter
12	**MM_0128**	M	NA	7	1	B	c.2521C>T	p.Gln841Ter
							c.6079C>T	p.Leu2027Phe
13	**MM_0129**	F	NA	12	NA	B	c.2521C>T	p.Gln841Ter
							c.6079C>T	p.Leu2027Phe
14	**MM_0130***	M	2186	8	1	B	c.768G>T	p.Val256Val,Splice site alteration
							c.634C>T	p.Arg212Cys
15	**MM_0131***	F	2186	7	1	B	c.768G>T	p.Val256Val,Splice site alteration
							c.634C>T	p.Arg212Cys
16	**MM_0138***	M	21220	7	3	B	c.5898+2T>C	Splice site alteration
							c.286A>T	p.Asn96Tyr
17	**MM_0146***	F	20522	14	1	B	c.5196+1137G>A	Deep intronic change
							c.3364G>A	p.Glu1122Lys
18	**MM_0148***	F	20026	8	3	B	c.5461-10T>C	Splice site alteration
							c.3259G>A	p.Glu1087Lys
19	**MM_0185***	F	20660	5	NA	B	c.4918C>T;c.4222T>C	p.Arg1640Trp;p.Trp1408Arg
							c.247_248insCAAA	p.Gln83ProfsTer17
20	**MM_0225***	M	22229	4	2	C	c.4469G>A	p.Cys1490Tyr
							c.6449G>A	p.Cys2150Tyr
21	**MM_0227***	F	32058	12	1	C	c.6391G>A	p.Glu2131Lys
							c.1957C>T	p.Arg653Cys
							c.1411G>A	p.Glu471Lys
22	**MM_0228***	F	32453	9	2	D	c.3113C>T;c.1622T>C	p.Ala1038Val;p.Leu541Pro
23	**MM_0230***	F	21352	7	1	C	c.4139C>T	p.Pro1380Leu
							c.2588G>C	p.Gly863Ala
24	**MM_0240***	M	21562	5	1	D	c.29_30insT	p.Leu10PhefsTer44
25	**MM_0241***	F	30455	6	3	C	c.3064G>A	p.Glu1022Lys
							c.3064G>A	p.Glu1022Lys
26	**MM_0246***	M	21513	7	3	C	c.5461-10T>C	Splice site alteration
							c.4326C>A	p.Asn1442Lys

Supplementary Table 1- Continue

Children at Baseline with Childhood-onset Disease

	Patient ID	Sex	Pedigree	Age of Disease Onset	ERG Group	Genotype Classification	Variants cDNA-Change	Protein Effect
27	**MM_0258***	F	31588	10	1	C	c.4793C>A	p.Ala1598Asp
							c.2300T>A	p.Val767Asp
28	**MM_0260***	F	20825	6	1	B	**c.4539+2065C>G**	**Deep intronic change**
							c.6118C>T	***p.Arg2040Ter***
29	**MM_0267***	M	19363	7	1	C	c.4469G>A	p.Cys1490Tyr
							c.5318C>A	p.Ala1973Glu
30	**MM_0281***	F	21433	4	3	B	c.4469G>A	p.Cys1490Tyr
							c.3191-1G>T	Splice site alteration
31	**MM_0282***	F	22725	6	3	B	c.5461-10T>C	Splice site alteration
							c.4363T>C	p.Cys1455Arg
32	**MM_0284***	M	21244	7	NA	C	c.1253T>C	p.Phe418Ser
							c.1253T>C	p.Phe418Ser
33	**MM_0286***	M	18989	9	2	C	c.4685T>C	p.Ile1562Thr
							c.4469G>A	p.Cys1490Tyr
							c.2588G>C	p.Gly863Ala
34	**MM_0291***	M	22635	10	1	C	c.1648G>A	p.Gly550Arg
							c.4918C>T	p.Arg1640Trp
35	**MM_0310***	M	22586	7	1	B	c.885delC	p.Asp295AspfsTer5
							c.1804C>T	p.Arg602Trp
36	**MM_0312***	F	22835	6	2	B	c.3322C>T	p.Arg1108Cys
							c.454C>T	p.Arg152Ter
37	**MM_0314***	F	20680	7	1	B	c.5461-10T>C	Splice site alteration
							c.4139C>T	p.Pro1380Leu
38	**MM_0324**	F	21659	10	1	D	c.4774-27T>C	Deep intronic change
							c.5196+1137G>A	Deep intronic change
39	**MM_0325***	F	21396	7	3	C	c.214G>A	p.Gly72Arg
							c.214G>A	p.Gly72Arg
40	**MM_0326***	M	21396	7	3	C	c.214G>A	p.Gly72Arg
							c.214G>A	p.Gly72Arg
41	**MM_0335***	F	21479	12	1	B	c.6319C>T	p.Arg2107Cys
							c.5461-10T>C	Splice site alteration
42	**MM_0351***	M	23342	8	1	B	c.1253T>C	p.Phe418Ser
							c.4773+1G>T	Splice site alteration
43	**MM_0356***	M	22496	7	1	C	c.1253T>C	p.Phe418Ser
							c.3322C>T	p.Arg1108Cys
44	**MM_0360***	F	21396	5	3	C	c.214G>A	p.Gly72Arg
							c.214G>A	p.Gly72Arg
45	**MM_0382***	F	22706	14	2	A	c.6445C>T	p.Arg2149Ter
							c.5018+5G>A	Splice site alteration
46	**MM_0399***	F	20236	11	1	B	c.1622T>C; c.3113C>T	p.Leu541Pro;p.Ala1038Val
							c.5714+5G>A	Splice site alteration
47	**MM_0421***	F	22362	11	3	C	c.4571A>G	p.Asp1524Gly
							c.4571A>G	p.Asp1524Gly
48	**MM_0426***	M	21140	6	1	A	c.6729+5_19delGTTGGCCCTGGGGCA	Splice site alteration
							c.6729+5_19delGTTGGCCCTGGGGCA	Splice site alteration
49	**MM_0431***	M	23221	13	2	C	c.6320G>A	p.Arg2107His
							c.2385C>G	p.Ser795Arg
50	**MM_0432***	M	22583	13	1	C	c.4462T>C	p.Cys1488Arg
							c.5882G>A	p.Gly1961Glu
51	**MM_0433***	M	32177	6	3	A	c.6729+5_19delGTTGGCCCTGGGGCA	Splice site alteration
							c.6729+5_19delGTTGGCCCTGGGGCA	Splice site alteration

Supplementary Table 1- Continue

Children at Baseline with Childhood-onset Disease

	Patient ID	Sex	Pedigree	Age of Disease Onset	ERG Group	Genotype Classification	Variants cDNA-Change	Protein Effect
52	**MM_0442**	F	22326	8	3	A	c.5904delT	p.Phe1968LeufsTer6
							c.5904delT	p.Phe1968LeufsTer6
53	**MM_0444**	F	25860	8	3	C	c.1622T>C	p.Leu541Pro
							c.3113C>T	p.Ala1038Val
							c.4469G>A	p.Cys1490Tyr
54	**MM_0454**	M	23153	10	1	C	c.4670A>G	p.Tyr1557Cys
							c.4670A>G	p.Val2050Leu
							c.4577C>T	p.Thr1526Met
55	**MM_0471**	M	23550	4	2	A	c.6729+5_19delGTTGGCCCTGGGGCA	Splice site alteration
							c.6729+5_19delGTTGGCCCTGGGGCA	Splice site alteration
56	**MM_0472**	F	26092	8	3	A	c.5917delG	p.Val1973Ter
							c.5917delG	p.Val1973Ter

Adults at Baseline with Childhood-onset Disease

	Patient ID	Sex	Pedigree	Age of Disease Onset	ERG Group	Genotype Classification	Variants cDNA-Change	Protein Effect
1	**MM_0065**	F	20842	16	3	B	c.5196+1G>T	Splice site alteration
							c.6079C>T	p.Leu2027Phe
2	**MM_0161**	M	20978	14	NA	C	c.3322C>T	p.Arg1108Cys
							c.5882G>A	p.Gly1961Glu
3	**MM_0242**	F	14447	14	1	B	c.6209C>G	p.Thr2070Arg
							c.5714+5G>A	Splice site alteration
4	**MM_0243**	F	14447	14	1	B	c.6209C>G	p.Thr2070Arg
							c.5714+5G>A	Splice site alteration
5	**MM_0247**	M	22853	14	1	C	c.2588G>C	p.Gly863Ala
							c.4918C>T	p.Arg1640Trp
6	**MM_0317**	F	21553	5	3	C	c.161G>A	p.Cys54Tyr
							c.3259G>A	p.Glu1087Lys
7	**MM_0332**	M	21868	10	3	B	c.5461-10T>C	Splice site alteration
							c.4577C>T	p.Thr1526Met
8	**MM_0336**	F	21479	14	1	B	c.5461-10T>C	Splice site alteration
							c.6319C>T	p.Arg2107Cys
9	**MM_0353**	M	23820	8	1	C	c.5882G>A	p.Gly1961Glu
							c.53G>A	p.Arg18Gln
10	**MM_0383**	F	23050	13	1	C	c.6079C>T	p.Leu2027Phe
							c.6079c>T	p.Leu2027Phe
11	**MM_0388**	F	20153	16	1	B	c.2588G>C	p.Gly863Ala
							c.2041C>T	p.Arg681Ter
12	**MM_0393**	F	20331	16	1	D	**c.3329-17T>G**	**Deep intronic change**
							c.5882G>A	p.Gly1961Glu
13	**MM_0403**	F	21830	13	1	B	c.1906C>T	p.Gln636Ter
							c.6079C>T	p.Leu2027Phe
14	**MM_0417**	M	25504	8	2	B	c.2588G>C	p.Gly863Ala
							c.4234C>T	p.Gln1412Ter
15	**MM_0448**	F	21519	13	1	B	c.3322C>T	p.Arg1108Cys
							c.1760+2T>C	Splice site alteration

Supplementary Table 1- Continue

	Patient ID	Sex	Pedigree	Age of Disease Onset	ERG Group	Genotype Classification	Variants cDNA-Change	Protein Effect
Adults at Baseline with Adulthood-onset Disease								
1	**MM_0019**	M	20341	32	1	C	c.6317G>A	p.Arg2106His
							c.6317G>A	p.Arg2106His
2	**MM_0025**	F	20573	30	3	C	c.4739T>C	p.Leu1580Ser
							c.2617T>C	p.Phe873Leu
3	**MM_0047**	M	20764	17	1	B	c.6729+5_19del15	p.Phe2161Cysfs*3
							c.4739T>C	p.Leu1580Ser
4	**MM_0057**	F	20801	36	1	C	***c.2522A>C***	***p.Gln841Pro***
							c.5882G>A	p.Gly1961Glu
5	**MM_0092**	F	17621	29	1	A	c.859-9T>C	Splice site alteration
							c.859-9T>C	Splice site alteration
6	**MM_0101**	M	20917	27	1	D	c.5882G>A	p.Gly1961Glu
7	**MM_0104**	M	19967	33	1	B	c.4637T>G	p.Leu1546Ter
							c.5882G>A	p.Gly1961Glu
8	**MM_0139**	F	19444	20	1	B	c.571-1G>T	Splice site alteration
							c.5087G>A	p.Ser1696Asn
9	**MM_0160**	M	21153	42	2	B	**c.5196+1216C>A**	**Deep intronic change**
							c.2588G>C	p.Gly863Ala
							**c.-14G>A**	**5'-UTR**
10	**MM_0318**	M	16487	18	1	C	c.6089G>A	p.Arg2030Gln
							c.4222T>C	p.Trp1408Arg
							c.4918C>T	p.Arg1640Trp
11	**MM_0333**	F	33458	28	1	C	c.2894A>G	p.Asn965Ser
							c.5882G>A	p.Gly1961Glu
12	**MM_0365**	M	16854	39	1	C	c.4577C>T	p.Thr1526Met
							c.71G>A	p. Arg24His
13	**MM_0376**	F	25338	25	1	B	***c.5931_5941dup11***	***p.Phe1982LysfsTer14***
							c.5882G>A	p.Gly1961Glu
14	**MM_0390**	F	25352	40	1	C	c.5882G>A	p.Gly1961Glu
							c.2297G>A	p.Gly766Asp
15	**MM_0409**	F	22973	35	1	C	c.6079C>T	p.Leu2027Phe
							c.1253T>C	p.Phe418Ser
16	**MM_0425**	F	31700	26	Normal	C	c.5882G>A	p.Gly1961Glu
							c.4793C>A	p.Ala1598Asp
17	**MM_0428**	M	35573	18	1	C	c.1253T>C	p.Phe418Ser
							c.2588G>C	p.Gly863Ala
18	**MM_0429**	M	16804	17	1	B	c.5882G>A	p.Gly1961Glu
							c.5461-10T>C	Splice site alteration
19	**MM_0465**	F	22054	26	1	C	c.5882G>A	p.Gly1961Glu
							c.5882G>A	p.Gly1961Glu

Pathogenicity of novel variants was assessed with the previously reported methods. (Fujinami et al. Invest Ophthalmol Vis Sci. 2013;54:6662–6674)
Novel possible disease-causing variants are in bold and italics
Novel variants wtih uncertain effects are in bold and underlined
***Patients previously reported; Tanna P, Georgiou M, Strauss RW, et al. Cross-Sectional and Longitudinal Assessment of the Ellipsoid Zone in Childhood-Onset Stargardt Disease. Transl Vis Sci Technol 2019;8(2):1. [36]**
