# Supplementary material for: Prospective Cohort Study of Childhood-Onset Stargardt Disease: Fundus Autofluorescence Imaging, Progression, Comparison with Adult-Onset Disease, and Disease Symmetry
Source: Am J Ophthalmol. 2020 Mar;211:159–75. doi: 10.1016/j.ajo.2019.11.008 (PMC7082771; doi:10.1016/j.ajo.2019.11.008)
Supplement: Supplemental Material 2 [file mmc2.pdf]

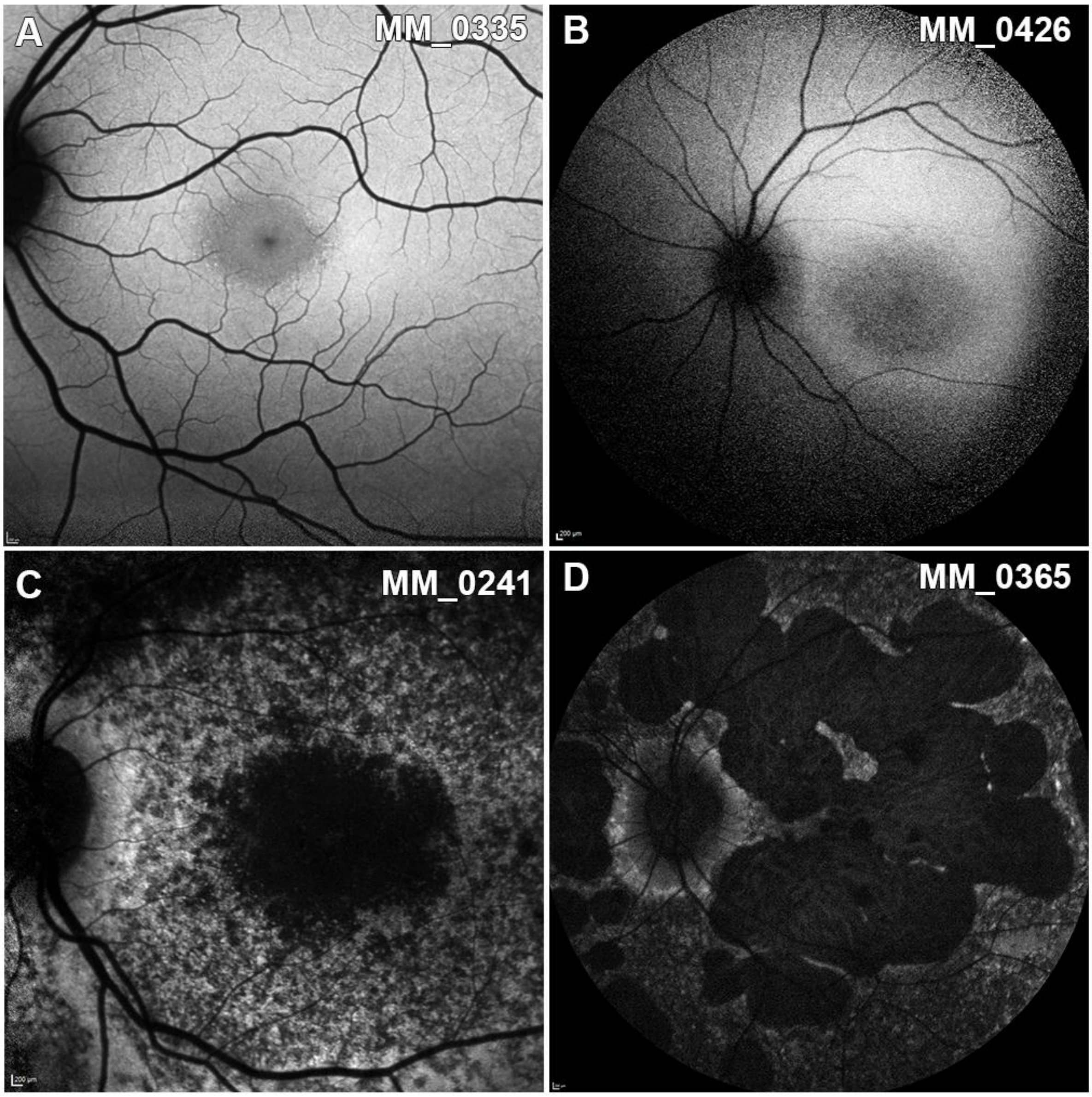

Supplementary Material 2: Examples of Images Excluded from Quantitative Analysis (SM1)

From the 360 images, 26 images from 10 subjects were excluded from further quantitative analysis. **(A-D)** examples of the excluded images. **(A)** Absence of area of Decreased autofluorescence (DAF). **(B)** Poor image quality. **(C)** and **(D)** area of DAF extending beyond borders of image.
